# Model Predictive Control with Variational Autoencoders for Signal Temporal Logic Specifications

**DOI:** 10.3390/s24144567

**Published:** 2024-07-14

**Authors:** Eunji Im, Minji Choi, Kyunghoon Cho

**Affiliations:** Department of Information and Telecommunication Engineering, Incheon National University, Incheon 22012, Republic of Korea; twintwin0243@inu.ac.kr (E.I.); if96e55@inu.ac.kr (M.C.)

**Keywords:** deep learning-based control synthesis, formal methods, rule-based path planning

## Abstract

This paper presents a control strategy synthesis method for dynamical systems with differential constraints, emphasizing the prioritization of specific rules. Special attention is given to scenarios where not all rules can be simultaneously satisfied to complete a given task, necessitating decisions on the extent to which each rule is satisfied, including which rules must be upheld or disregarded. We propose a learning-based Model Predictive Control (MPC) method designed to address these challenges. Our approach integrates a learning method with a traditional control scheme, enabling the controller to emulate human expert behavior. Rules are represented as Signal Temporal Logic (STL) formulas. A robustness margin, quantifying the degree of rule satisfaction, is learned from expert demonstrations using a Conditional Variational Autoencoder (CVAE). This learned margin is then applied in the MPC process to guide the prioritization or exclusion of rules. In a track driving simulation, our method demonstrates the ability to generate behavior resembling that of human experts and effectively manage rule-based dilemmas.

## 1. Introduction

Robotics is increasingly permeating diverse sectors, spanning both civilian and industrial applications, and is becoming integral to everyday life. Service robots are now prevalent in public spaces, interacting with individuals and delivering services. Within the field of robotics, autonomous driving emerges as a particularly dynamic area, garnering extensive research attention.

In robotics, adherence to rules varies from basic collision avoidance in navigation scenarios to compliance with complex traffic regulations in autonomous driving. These rules, established primarily for safety, must generally be upheld by robots while executing their tasks. However, it is essential to acknowledge that not all rules carry equal importance. Depending on the context, some rules may need to be prioritized over others or even disregarded. For example, in autonomous driving, scenarios may necessitate breaching certain rules—such as lane changes in dense traffic, decisions at yellow traffic lights, or crossing double yellow lines to avoid obstacles. These situations compel robots to make intricate decisions regarding rule compliance, presenting significant challenges in determining appropriate control inputs.

Model Predictive Control (MPC) stands out as a robust approach for autonomous control, recognized for its capabilities in online trajectory optimization [1]. The core principle of MPC involves identifying optimal control inputs to minimize a predefined cost function, considering both inputs and anticipated future outputs. This method integrates an objective function characterizing the desired robot behavior and constraints mitigating undesirable actions. The efficacy of MPC is well-documented across diverse applications, such as the full-body control of humanoid robots [2,3,4].

Designing effective MPC controllers remains a significant challenge. Experienced operators can adeptly manage robots, yet encoding such expertise into MPC parameters is complex. For instance, expert drivers in autonomous driving must make continuous, complex decisions, such as whether to decelerate or change lanes in response to slow-moving vehicles. However, finding the appropriate MPC parameters to handle such varied scenarios is complex and computationally intensive.

Recently, imitation learning has emerged as a promising solution for robotic learning challenges [5,6]. This approach derives near-optimal control strategies directly from human expert demonstrations, eliminating the need for manual policy or cost function design. Imitation learning excels in capturing complex policy functions that balance multiple considerations [7], learning the importance of various factors from expert behaviors to enable robots to replicate human actions. However, despite its advantages, imitation learning does not inherently ensure performance reliability. In scenarios where safety rules like collision avoidance are crucial, imitation learning may not consistently yield control actions that comply with essential safety norms, underscoring the paramount importance of rule adherence for robot safety and human protection.

In this paper, we address a control synthesis problem within a framework of prioritized rules, building on our previous research [8]. We assumed inherent rule priorities and aimed to design a controller that accounts for these priorities to manage dilemmas effectively. Our methodology is grounded in the MPC framework, which, unlike purely deep learning-based approaches, integrates each rule as a constraint, thereby enhancing performance reliability.

We represent these rules using Signal Temporal Logic (STL) [9,10], a formalism allowing the precise specification of desired system behaviors, commonly applied in robotic task specifications [11,12,13,14,15]. STL is particularly suited for describing properties of real-valued signals in dense time scenarios, making it ideal for real-world robotic applications.

Instead of explicitly determining rule priorities, we adopted a learning approach to identify minimal acceptable levels of rule satisfaction, informed by expert demonstrations. This approach diverges from our earlier work [8] by employing a Conditional Variational Autoencoder (CVAE) [16]. This technique helps discern essential rules and decide on adherence levels, facilitating selective compliance rather than strict obedience to all rules. The use of a CVAE is justified by its efficiency in handling uncertainties within data, providing a more effective solution compared to Gaussian process regression methods used in previous work [8].

Our hybrid approach combines deep learning with traditional MPC, guiding robots to emulate expert human behaviors in complex decision-making scenarios.

## 2. Related Work

Extensive research has explored trajectory optimization and Model Predictive Control (MPC) within the framework of temporal logic specifications, particularly Linear Temporal Logic (LTL). Mixed-integer linear programming (MILP) has been employed to generate trajectories for continuous systems subject to finite-horizon LTL specifications [17,18]. Wolff et al. [19] extended this approach by encoding general LTL formulas into MILP constraints, accommodating infinite runs with periodic structures. Additionally, Cho et al. [20] investigated optimal path planning under synthetically co-safe LTL specifications, utilizing a sampling-tree and two-layered structure.

Recent advancements have integrated Signal Temporal Logic (STL) within MPC frameworks. Raman et al. [21] structured MPC to facilitate control synthesis from STL specifications using MILP, allowing for the calculation of open-loop control signals that adhere to both finite and infinite horizon STL properties while maximizing robust satisfaction. Sadigh et al. [22] introduced a novel STL variant incorporating probabilistic predicates to address uncertainties in predictive models, thereby enhancing safety assessments under uncertainty. Mao et al. [23] proposed a solution to handle complex temporal requirements formalized in STL specifications within the Successive Convexification algorithmic framework. This approach retains the expressiveness of encoding mission requirements with STL semantics while avoiding combinatorial optimization techniques such as MILP.

The integration of MPC with machine learning techniques has been pursued to address system identification challenges within MPC contexts [24,25,26]. Lenz et al. [24] applied deep learning within MPC to derive task-specific controls for complex activities such as robotic food cutting. Carron et al. [25] presented a model-based control approach that utilizes data gathered during operation to improve the model of a robotic arm and thereby enhance the tracking performance. Their scheme is based on inverse dynamic feedback linearization and a data-driven error model, integrated into an MPC formulation. Lin et al. [26] compared deep reinforcement learning (DRL) and MPC for Adaptive Cruise Control (ACC) design in car-following scenarios.

Efforts have also been made to address the types of dilemmas introduced in our work. Tumova et al. [27] and Castro et al. [28] examined scenarios where not all LTL rules can be satisfied in path planning, seeking paths that minimally violate these rules. However, their approaches require predetermined weights among rules, contrasting with our method that learns directly from expert demonstrations. Urban driving dilemmas were specifically addressed by Lee et al. [29], who applied inverse reinforcement learning to capture expert driving strategies.

Imitation learning is emerging as a promising approach to robotic learning problems and has been widely applied to autonomous driving. Policies for autonomous vehicles have been learned from image or video datasets through Convolutional Neural Networks (CNNs) [30,31]. Schmerling et al. [32] utilized a Conditional Variational Autoencoder (CVAE) framework to reason about interactions between vehicles in traffic-weaving scenarios, producing multimodal outputs. Additionally, some studies have applied learning approaches to MPC, where certain parameters of MPC are learned from data [8,33]. Reinforcement learning has also been considered for autonomous driving, using CNNs to encode visual information [34].

## 3. Preliminaries

### 3.1. System Model

We consider a continuous-time dynamical system described by the following differential equation:

(1) $${\dot{x}}_{t}=f\left(x_{t},u_{t}\right),$$

where $x_{t}\in X\subset R^{n_{x}}$ represents the state vector, $u_{t}\in U\subset R^{n_{u}}$ denotes the control input, and *f* is a smooth (continuously differentiable) function with respect to its arguments. Through employing a predefined time step dt, the continuous system in Equation (1) can be discretized as follows:

(2) $$x_{n+1}=f\left(x_{n},u_{n}\right),$$

where *n* represents the discrete time step, defined as n=⌊t/dt⌋, and $x_{0}$ denotes the initial state. For a fixed horizon *H*, let $x(x_{n},u^{H,n})$ denote a trajectory generated from the state $x_{n}$ with the control inputs $u^{H,n}=\left\{u_{n},\ldots ,u_{n+H-1}\right\}$.

A *signal* is defined as a sequence of states and control inputs:

(3) $$\xi \left(x_{n},u^{H,n}\right)=\left(x_{n},u_{n}\right),\ldots ,\left(x_{n+H-1},u_{n+H-1}\right).$$

In addition to the definition provided in Equation (3), we use the notation ξ(n) to represent a signal starting from the discrete time step *n*, with a slight abuse of notation.

### 3.2. Signal Temporal Logic

Signal Temporal Logic (STL) is a formalism used to specify properties of real-valued, dense-time signals, and is extensively applied in the analysis of continuous and hybrid systems [9,10]. A predicate within an STL formula is defined as an inequality of the form μ(ξ(t))>0, where μ is a function of the signal ξ at time *t*. The truth value of the predicate μ is determined by the condition μ(ξ(t))>0.

An STL formula is composed of boolean and temporal operations on these predicates. The syntax of STL formulae φ is defined recursively as follows:

(4) $$\varphi ::=\mu \;|\;\neg \mu \;|\;\varphi \wedge \psi \;|\;G_{\left[a,b\right]}\psi \;|\;\varphi U_{\left[a,b\right]}\psi ,$$

where φ and ψ are STL formulas, G denotes the *globally* operator, and U represents the *until* operator.

The validity of an STL formula φ with respect to a signal ξ at time *t* is defined inductively as follows:

(5) (ξ,t)⊧μ⇔μ(ξ(t))>0

(6) (ξ,t)⊧¬μ⇔¬((ξ,t)⊧μ)

(7) (ξ,t)⊧φ∧ψ⇔(ξ,t)⊧φ∧(ξ,t)⊧ψ

(8) (ξ,t)⊧φ∨ψ⇔(ξ,t)⊧φ∨(ξ,t)⊧ψ

(9) $$\left(\xi ,t\right)⊧G_{\left[a,b\right]}\varphi \Leftrightarrow \forall t^{\prime }\in \left[t+a,t+b\right],\;\left(\xi ,t^{\prime }\right)⊧\varphi$$

(10) $$\left(\xi ,t\right)⊧\varphi U_{\left[a,b\right]}\psi \Leftrightarrow \exists t^{\prime }\in \left[t+a,t+b\right]\;s.t.\left(\xi ,t^{\prime }\right)⊧\psi \;\wedge \forall t^{\prime \prime }\in \left[t,t^{\prime }\right],\;\left(\xi ,t^{\prime \prime }\right)⊧\varphi .$$

The notation (ξ,t)⊧φ indicates that the signal ξ satisfies the STL formula φ at time *t*. For example, $\left(\xi ,t\right)⊧G_{\left[a,b\right]}\varphi$ implies that φ holds for the signal ξ throughout the interval from t+a to t+b. In discrete-time systems, STL formulas are evaluated over discrete time intervals.

One significant advantage of Signal Temporal Logic (STL) is its associated metric, known as the *robustness degree*, which quantifies how well a given signal ξ satisfies an STL formula φ. The robustness degree is defined as a real-valued function of the signal ξ and time *t*, calculated recursively using the following *quantitative semantics*:

(11) $$\begin{array}{cccc} \; & \rho^{\mu }\left(\xi ,t\right) & = & \mu (\xi (t)), \end{array}$$

(12) $$\begin{array}{cccc} \; & \rho^{\neg \mu }\left(\xi ,t\right) & = & -\mu (\xi (t)), \end{array}$$

(13) $$\begin{array}{cccc} \; & \rho^{\varphi \wedge \psi }\left(\xi ,t\right) & = & \min(\rho^{\varphi }\left(\xi ,t\right),\rho^{\psi }\left(\xi ,t\right)), \end{array}$$

(14) $$\begin{array}{cccc} \; & \rho^{\varphi \vee \psi }\left(\xi ,t\right) & = & \max(\rho^{\varphi }\left(\xi ,t\right),\rho^{\psi }\left(\xi ,t\right)), \end{array}$$

(15) $$\begin{array}{cccc} \; & \rho^{G_{\left[a,b\right]}\varphi }\left(\xi ,t\right) & = & \min_{t^{\prime }\in \left[t+a,t+b\right]}\rho^{\varphi }\left(\xi ,t^{\prime }\right), \end{array}$$

(16) $$\begin{array}{cccc} \; & \rho^{\varphi U_{\left[a,b\right]}\psi }\left(\xi ,t\right) & = & \max_{t^{\prime }\in \left[t+a,t+b\right]}(\min(\rho^{\psi }\left(\xi ,t^{\prime }\right), \end{array}$$

(17) $$\begin{array}{cccc} \; & \; & & \min_{t^{\prime \prime }\in \left[t,t^{\prime }\right]}\rho^{\varphi }\left(\xi ,t^{\prime \prime }\right))). \end{array}$$

Following our previous study [8], we introduce the notation (ξ,t)⊧(φ,r) to indicate that the signal ξ satisfies the STL formula φ at time *t* with a *robustness slackness r*, defined as

(18) $$\left(\xi ,t\right)⊧\left(\varphi ,r\right)\;\;\;\equiv \;\;\;\rho^{\varphi }\left(\xi ,t\right)>r.$$

Equation (18) asserts that the signal ξ satisfies φ with at least the minimum robustness degree *r*. The robustness slackness *r* serves as a margin for the satisfaction of the STL formula φ. As *r* increases, the constraints on the signal ξ to satisfy φ at time *t* become more stringent, while smaller values of *r* imply more relaxed constraints. Notably, when r<0, it allows for the violation of φ.

## 4. Problem Formulation

This study aimed to solve a control synthesis problem using Signal Temporal Logic (STL) formulas [8]. Let $\varphi =[\varphi_{1},\ldots ,\varphi_{N}]$ represent a set of STL formulas, with their conjunction denoted as $\bar{\varphi }=\varphi_{1}\wedge \ldots \wedge \varphi_{N}$. We define a cost function *J* over the state and control spaces, where J(x,u) measures the cost associated with a trajectory x and control sequence u. The control synthesis problem under STL for Model Predictive Control (MPC) is formulated as follows.

**Problem** **1.** *Given a system model as described in (2) and an initial state $x_{0}$, with a planning horizon of length H, determine the control input sequence $u^{H,t}$ at each time step t that minimizes the cost function $J(x\left(x_{t},u^{H,t}\right),u^{H,t})$ while ensuring that the conjunction of STL formulas $\bar{\varphi }$ is satisfied:*

(19) $$\begin{array}{cccc} \; & \underset{u^{H,t}}{\mathrm{minimize}}\; & \; & J(x\left(x_{t},u^{H,t}\right),u^{H,t})\\ \; & \mathrm{subject}\;\mathrm{to} & \; & \left(\xi \left(x_{t},u^{H,t}\right),t\right)⊧\bar{\varphi }. \end{array}$$

While this strict formulation ensures compliance with the STL formulas, our primary objective is to develop a control sequence that incorporates flexibility in rule compliance. To this end, we introduce robustness slackness values, denoted by $r=[r_{1},\ldots ,r_{N}]$, which quantify the degree to which each STL formula is satisfied. In incorporating these robustness values, the MPC problem can be reformulated as follows [8].

**Problem** **2.** *Given the system model specified in (2), an initial state $x_{0}$, and a horizon length H, compute the control input sequence $u^{H,t}$ at each time step t by solving the following optimization problem:*

(20) $$\begin{array}{cccc} \; & \underset{u^{H,t}}{\mathrm{minimize}}\; & \; & J(x\left(x_{t},u^{H,t}\right),u^{H,t})\\ \; & \mathrm{subject}\;\mathrm{to} & \; & \left(\xi \left(x_{t},u^{H,t}\right),t\right)⊧\left(\varphi_{1},r_{1}\right),\\ \; & \; & & \;\;\;\;\vdots \\ \; & \; & & \left(\xi \left(x_{t},u^{H,t}\right),t\right)⊧\left(\varphi_{N},r_{N}\right). \end{array}$$

This enhanced formulation allows for a more flexible management of STL constraints, effectively addressing scenarios where it is not feasible to fully satisfy all STL formulas. The robustness slackness values are derived from expert demonstrations, based on the assumption that these experts have accurately assessed the priority and required compliance level of each rule. This learning is achieved through a deep learning approach.

## 5. Proposed Method

**Figure 1:**
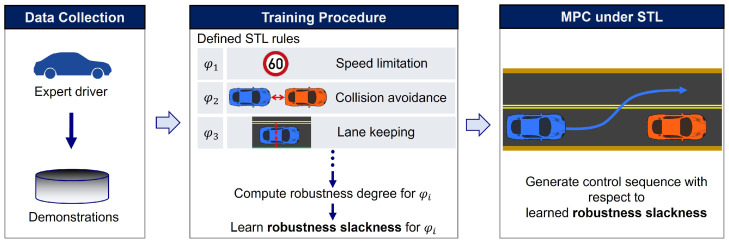
Overview of the proposed learning-based MPC framework. Expert demonstrations are utilized to learn the lower bounds of robustness, referred to as robustness slackness, through a deep learning approach. The learned values inform the MPC method, which then computes control sequences considering the STL rules.

The proposed framework, illustrated in Figure 1, synergizes learning techniques with STL constraints to refine MPC, enabling it to more accurately mimic human expert behavior. By leveraging expert demonstrations, we learn robustness slackness values, which define the margins of rule compliance. A Conditional Variational Autoencoder (CVAE) [16] is utilized to estimate these robustness slackness values in novel scenarios.

In incorporating the robustness slackness values obtained through the learning process, the MPC method, designed under STL constraints, generates control sequences that respect the specified rules with a certain level of flexibility. To manage the nonlinear differential constraints characteristic of dynamical systems, we employ linearized models. Although this approach may introduce some approximation errors, it remains effective for practical applications.

Figure 1 presents an overview of the proposed learning-based MPC framework. Expert demonstrations are used to learn the lower bounds of robustness, referred to as robustness slackness, through a deep learning approach. These learned values inform the MPC method, which then calculates control sequences that take into account the STL rules.

### 5.1. Feature Description

We introduce a feature function, denoted as ϕ, which transforms a signal into a feature vector, mapping from the combined state and control spaces into the feature space: $\phi :R^{n_{x}+n_{u}}\to R^{n_{f}}$.

**Figure 2:**
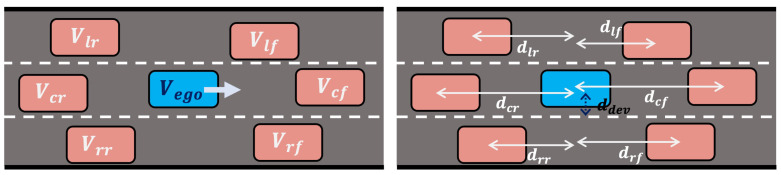
Description of the ego vehicle and nearby vehicles in a track driving scenario. The ego vehicle ($V_{ego}$) is shown in blue. The diagram includes up to six nearby vehicles positioned in front and behind, across the left, center, and right lanes relative to the ego vehicle.

As illustrated in Figure 2, the control of the ego vehicle, $V_{ego}$, is influenced by six nearby vehicles located in adjacent lanes. These vehicles are collectively referred to as $V_{near}=\left\{V_{lf},V_{lr},V_{cf},V_{cr},V_{rf},V_{rr}\right\}$, where the subscripts denote the relative position to the ego vehicle: left-front (lf), left-rear (lr), center-front (cf), center-rear (cr), right-front (rf), and right-rear (rr).

The feature vector ϕ includes the following components:

- Distances to each of the nearby vehicles ($d_{lf},d_{lr},d_{cf},d_{cr},d_{rf},d_{rr}$);
- Lateral deviation from the lane center ($d_{dev}$);
- Heading angle relative to the lane direction ($\theta_{dev}$).

### 5.2. Learning Robustness Slackness from Demonstration

We consider a set of *M* demonstrated signals, denoted by $\Xi ={\left\{\xi^{i}\right\}}_{i=1}^{M}$, where each signal $\xi_{n}^{i}=\left(x_{n}^{i},u_{n}^{i}\right)$ comprises the state $x_{n}^{i}$ and control input $u_{n}^{i}$ at time step *n*. The robustness degree $r^{i,j}$ is defined as the minimum value observed from the current time step *n* to the future time step n+H−1 for the demonstration $\xi^{i}$:

(21) $$r_{n}^{i,j}=\min_{m\in [n,n+H-1]}\rho^{\varphi_{j}}\left(\xi^{i},m\right),$$

where *H* denotes the control horizon length. The robustness degree $r^{i,j}$ serves as the robustness slackness for the signal over the horizon length *H*, starting from $\xi_{n}^{i}$, indicating the minimum permissible lower bound of robustness within this timeframe.

**Figure 3:**
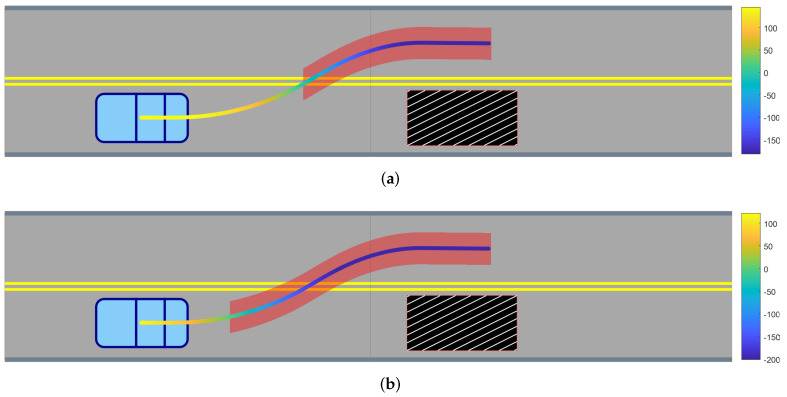
Demonstration in a track driving environment showing (**a**) the robustness degree and (**b**) its predicted lower bound. Trajectories with values less than zero are shaded in red.

Figure 3 illustrates a demonstrated trajectory in a track driving scenario, depicting both the robustness degree and its lower bound for the time horizon *H*. The rule considered involves maintaining the first (lowest) lane, defined by the STL formula $\varphi_{\mathrm{lane}}=\left(y\leq y_{\mathrm{upper}}\right)\wedge \left(y\geq y_{\mathrm{lower}}\right)$, where *y* represents the vertical position of the vehicle, and $y_{\mathrm{upper}}$ and $y_{\mathrm{lower}}$ are the upper and lower lane boundaries, respectively. An obstacle (or other vehicle), depicted as a striped black box, necessitates a lane change to proceed. The figure illustrates the variance between the robustness degree values and their corresponding lower bounds.

**Figure 4:**
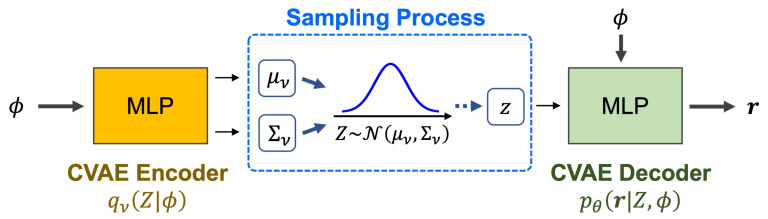
The CVAE network used to predict the robustness slackness.

From the demonstrated signals Ξ, let $D^{j}$ represent the outputs from Equation (21) corresponding to the STL formula $\varphi_{j}$. We define Φ as the set of feature vectors derived from the demonstrated signals Ξ (see Figure 2). For a new input feature ϕ, the CVAE network, depicted in Figure 4, predicts the lower bound of the robustness degree for the horizon *H*, representing the learned robustness slackness $r=[r_{1},\ldots ,r_{N}]$.

Our CVAE model comprises the following three parameterized functions:

- The **recognition model** $q_{\nu }\left(Z|\phi \right)$ approximates the distribution of the latent variable *Z* based on the input features. This is modeled as a Gaussian distribution, $N(\mu_{\nu }\left(\phi \right),\Sigma_{\nu }\left(\phi \right))$, where $\mu_{\nu }$ and $\Sigma_{\nu }$ represent the mean and covariance determined by the network.
- The **prior model** $p_{\theta }\left(Z|\phi \right)$ assumes a standard Gaussian distribution, N(0,I), simplifying the structure of the latent space.
- The **generation model** $p_{\theta }\left(r|Z,\phi \right)$ calculates the likelihood of robustness slackness based on the latent variable *Z* and the input feature ϕ.

Both the recognition model $q_{\nu }\left(Z|\phi \right)$ and the generation model $p_{\theta }\left(r|Z,\phi \right)$ are implemented as multi-layer perceptrons.

The training of our CVAE is guided by the Evidence Lower Bound (ELBO) loss function, initially formulated as

(22) $$E_{q_{\nu }\left(Z|\phi \right)}\left[\log p_{\theta }\left(r|Z,\phi \right)\right]-D_{KL}\left(q_{\nu }\left(Z|\phi \right)∥p_{\theta }\left(Z|\phi \right)\right).$$

To better accommodate the specific requirements of our application, we adapted the ELBO function and define the loss function as follows:

(23) $$-\sum_{i=1}^{N}\log\left(p_{\theta }\left(r_{i}|Z,\phi \right)\right)+\lambda \cdot D_{KL}\left(N(\mu_{\nu }\left(\phi \right),\Sigma_{\nu }\left(\phi \right))∥N\left(0,I\right)\right),$$

where $r_{i}$ represents an element of the robustness slackness r, and λ is a scaling factor used to balance the terms. The Kullback–Leibler divergence ($D_{KL}$) measures the divergence between two probability distributions. We set λ=1 and optimize parameters ν and θ by minimizing this loss function.

### 5.3. Model Predictive Control Synthesis

Previous work, such as that by Raman et al. [21], has shown that MPC optimization with STL constraints can be formulated as a mixed-integer linear program (MILP). This method introduces two encoding strategies: one that focuses on satisfying STL formulas and another, termed ‘robustness-based encoding’, that considers the robustness degree of the STL formulas. In our problem formulation, we manage each STL formula according to its defined robustness slackness using the robustness-based encoding method.

Let $C_{\varphi_{j},r_{j}}$ denote the encoded constraints for the STL formula $\varphi_{j}$ with robustness slackness $r_{j}$. The combined encoded constraints are formulated as follows:

(24) $$\begin{array}{cc} z^{\varphi }=\overset{N}{\underset{j=1}{⋀}}z^{\varphi_{j}}⟺ & \;z^{\varphi }\leq z^{\varphi_{j}}, \end{array}$$

(25) $$\begin{array}{cc} \; & \;z^{\varphi }\geq 1-N+\sum_{j}^{N}z^{\varphi_{j}}, \end{array}$$

where $z^{\varphi },z^{\varphi_{j}}\in \left[0,1\right]$ are Boolean variables, with $z^{\varphi }$ representing the satisfaction of all STL constraints and $z^{\varphi_{j}}$ representing the satisfaction of an individual STL formula $\varphi_{j}$. Note that $z^{\varphi_{j}}=1$ only if $\rho^{\varphi_{j}}-r_{j}>0$; otherwise, $z^{\varphi_{j}}=0$.

The proposed algorithm is outlined in Algorithm 1. We extended our previous work [8] by incorporating a deep learning network approach. Inputs to the algorithm include a set of STL formulas $\varphi_{1},\ldots ,\varphi_{N}$, the time of interest $\tau =[t_{0},t_{1}]$, the discretization time step dt, a control horizon *H*, an initial signal state $\xi_{init}$, and demonstrated signals Ξ.

Initially, feature vectors and robustness slackness values (the lowest robustness degree for the horizon *H*) are pre-computed from demonstrations (line 1). The closed-loop algorithm, which determines the optimal strategy at each time step, runs over the time interval $\tau =[t_{0},t_{1}]$. Nonlinear dynamics are linearized with respect to the current signal state (line 4). The robustness slackness of the STL formula $\varphi_{j}$ for the input feature $\phi (\xi_{cur})$ is predicted using the trained CVAE network (line 6). The predicted robustness slackness for each STL formula is denoted as $r_{j}$. Based on the updated robustness slackness $r_{j}$, each STL formula $\varphi_{j}$ is converted into mixed-integer programming constraints $C_{\varphi_{j},r_{j}}$ using the robustness-based encoding method (line 7), where $C_{\varphi_{j},r_{j}}$ consists of binary variables and linear predicates. In considering all STL constraints, dynamic constraints, and past trajectories, the optimal control sequence is computed over the time horizon *H* using a user-defined cost function (line 11). This procedure is repeated for the entire time interval τ.
**Algorithm 1** Variational Autoencoder-based Controller Synthesis under STL Constraints  1:$\Phi ,D^{j}\leftarrow$ Initialize(Ξ)  2:$\xi_{cur}\leftarrow \xi_{init},\;\xi_{past}\leftarrow \emptyset$  3:**for** $t=t_{0}:dt:t_{1}$ **do**  4:     $f_{lin}\leftarrow$ Linearize($f,\xi_{cur}$)  5:     **for** j=1:1:N **do**  6:          $r_{j}\leftarrow$ CVAE($\phi (\xi_{cur})$)  7:          $C_{\varphi_{j},r_{j}}\leftarrow$ EncodeSTLConstraints($\varphi_{j},r_{j}$)  8:     **end for**  9:     $C_{STL}\leftarrow C_{\varphi_{1},r_{1}}\wedge \ldots \wedge C_{\varphi_{N},r_{N}}$10:     $C\leftarrow C_{STL}\wedge f_{lin}\wedge \left[\xi \left(t_{0},\cdots ,t-dt\right)=\xi_{past}\right]$11:     $u^{H,t}\leftarrow$ Optimize($J(\xi^{H}),C$)12:     $x_{next}=f\left(x_{cur},u^{H,t}\left(t\right)\right)$13:     $\xi_{past}\leftarrow \left[\xi_{past}\;\xi_{cur}\right]$14:     $\xi_{cur}\leftarrow \left(x_{next},u^{H,t}\left(t\right)\right)$15:**end for**

## 6. Experimental Results

The proposed algorithm was implemented in a Python (version 3.10) environment, utilizing PyTorch (version 2.2.1) [35] for the deep learning components and Gurobi [36] as the optimization engine for MPC. Simulation experiments were conducted on a system equipped with an AMD R7-7700 processor and an RTX 4080 Super GPU. The Gurobi tool enabled solving the proposed MPC problem in approximately 0.11 s.

We conducted realistic simulations using the Next Generation Simulation (NGSIM) dataset [37] and the highD dataset [38], assuming that the drivers in these datasets possessed a certain level of expertise, making them suitable for “expert driver” demonstrations in our proposed approach. In the proposed method, obstacles were set as nearby vehicles. For generating training data, we utilized a combination of 70% data from the highD dataset and 30% from the NGSIM dataset. Data points from the NGSIM dataset that involved vehicles deviating from the track or causing collisions were excluded or modified. Additionally, data with normal speeds but no lane changes were partially removed to ensure a diverse set of training scenarios.

The CVAE network was trained with the following hyperparameters: a batch size of 64, a learning rate of 0.001, and 100 epochs. The future time horizon *H* was set to 16.

### 6.1. System Description

We modeled the dynamics of the vehicles on the track using a unicycle model. The state of the system at time *t* is described by $x_{t}={\left[x_{t},y_{t},\theta_{t},v_{t}\right]}^{T}$, where $x_{t}$ and $y_{t}$ represent the vehicle’s position, $\theta_{t}$ denotes the heading angle, and $v_{t}$ indicates the linear velocity. The control inputs are $u_{t}={\left[w_{t},a_{t}\right]}^{T}$, with $w_{t}$ as the angular velocity and $a_{t}$ as the acceleration. The vehicle dynamics are expressed as follows:

$$\begin{array}{c} {\dot{x}}_{t}=v_{t}\cos\left(\theta_{t}\right),\;{\dot{y}}_{t}=v_{t}\sin\left(\theta_{t}\right),\;{\dot{\theta }}_{t}=\kappa_{1}w_{t},\;{\dot{v}}_{t}=\kappa_{2}a_{t}, \end{array}$$

where $\kappa_{1}$ and $\kappa_{2}$ are constants. To facilitate the optimization process, we linearize the dynamics around a reference point $\hat{x}={\left[\hat{x},\hat{y},\hat{\theta },\hat{v}\right]}^{T}$. The resulting linear system is derived as a first-order Taylor approximation of the nonlinear dynamics, given by

$$\begin{array}{c} x_{n+1}=A_{n}x_{n}+B_{n}u_{n}+C_{n}, \end{array}$$

where the matrices $A_{n}$, $B_{n}$, and $C_{n}$ are defined as

$$\begin{array}{cc} A_{n} & =\left[\begin{array}{cccc} 1 & 0 & -\hat{v}\sin\left(\hat{\theta }\right)\;dt & \cos(\hat{\theta })\;dt\\ 0 & 1 & \hat{v}\cos\left(\hat{\theta }\right)\;dt & \sin(\hat{\theta })\;dt\\ 0 & 0 & 1 & 0\\ 0 & 0 & 0 & 1 \end{array}\right],\\ B_{n} & =\left[\begin{array}{cc} 0 & 0\\ 0 & 0\\ \kappa_{1}\hat{v}\;dt & 0\\ 0 & \kappa_{2}\;dt \end{array}\right],\\ C_{n} & =\left[\begin{array}{c} \hat{v}\sin\left(\hat{\theta }\right)\;\hat{\theta }\;dt\\ -\hat{v}\cos\left(\hat{\theta }\right)\;\hat{\theta }\;dt\\ 0\\ 0 \end{array}\right]. \end{array}$$

### 6.2. Rule Description

We formulated five distinct rules as STL formulas. The definitions of the rules $\varphi =[\varphi_{1},\ldots ,\varphi_{5}]$ are as follows:

1. Lane keeping (right): $\varphi_{1}=y_{t}\geq y_{l,\min}$;
2. Lane keeping (left): $\varphi_{2}=y_{t}\leq y_{l,\max}$;
3. Collision avoidance (front vehicle):
  $$\varphi_{3}=\left(x_{t}\leq x_{c,\min}\right)\vee \left(x_{t}\geq x_{c,\max}\right)\vee \left(y_{t}\leq y_{c,\min}\right)\vee \left(y_{t}\geq y_{c,\max}\right);$$
4. Speed limit: $\varphi_{4}=v_{t}\leq v_{th}$;
5. Slow down before the front vehicle:
  $$\varphi_{5}=\left(v_{t}\leq v_{u}\right)\;U_{\left[t_{a},t_{b}\right]}\;\left(x_{t}\leq x_{c,\min}\right).$$

In these formulations, $t_{a}$ and $t_{b}$ are set to 6 and 12, respectively.

**Figure 5:**
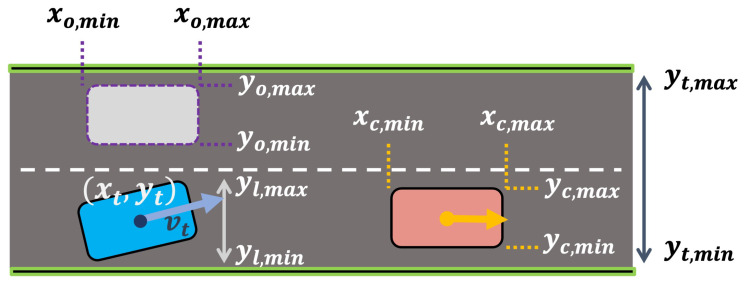
Driving environment illustrating the defined STL rules φ.

Figure 5 illustrates the driving environment used to describe these STL rules. Note that in this figure, the ego vehicle is depicted in blue, the preceding vehicle in orange, and other vehicles in gray. The positions $x_{t}$ and $y_{t}$ and velocity $v_{t}$ correspond to the ego vehicle. The boundaries of the preceding vehicle at the *x*-*y* coordinates are denoted by $x_{c,\min}$, $x_{c,\max}$, $y_{c,\min}$, and $y_{c,\max}$. Similarly, $x_{o,\min}$, $x_{o,\max}$, $y_{o,\min}$, and $y_{o,\max}$ represent the boundaries of other vehicles except the preceding one. The lane boundaries are denoted by $y_{l,\min}$ and $y_{l,\max}$, while the track boundaries are represented by $y_{t,\min}$ and $y_{t,\max}$.

Here, $v_{th}$ represents the speed limit threshold for rule $\varphi_{4}$. The final rule, $\varphi_{5}$, mandates that the ego vehicle decelerate when approaching a preceding vehicle in the same lane. Parameters $v_{u}$, $t_{a}$, and $t_{b}$ are specific to rule $\varphi_{5}$.

### 6.3. Simulation Results

**Figure 6:**
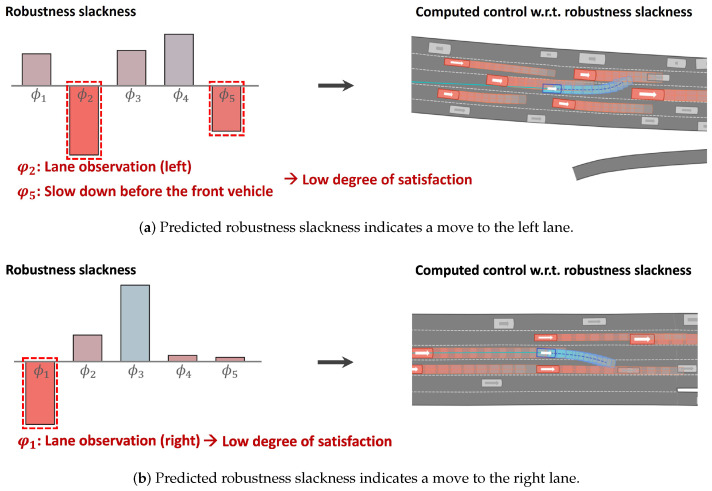
Snapshots of the proposed method applied to the NGSIM dataset.

Figure 6 presents the predicted robustness slackness r generated by the proposed CVAE network, alongside the control sequence produced by the MPC based on these predicted values. In the left subfigures indicating robustness slackness, negative degrees of satisfaction are marked with a red box.

In Figure 6a, the predicted robustness slackness suggests that rules $\varphi_{2}$ and $\varphi_{5}$ may be violated. It can be observed that the control sequence generated by the MPC results in the vehicle moving to the left lane (violating $\varphi_{2}$) and accelerating in the presence of a preceding vehicle (violating $\varphi_{5}$).

**Figure 7:**
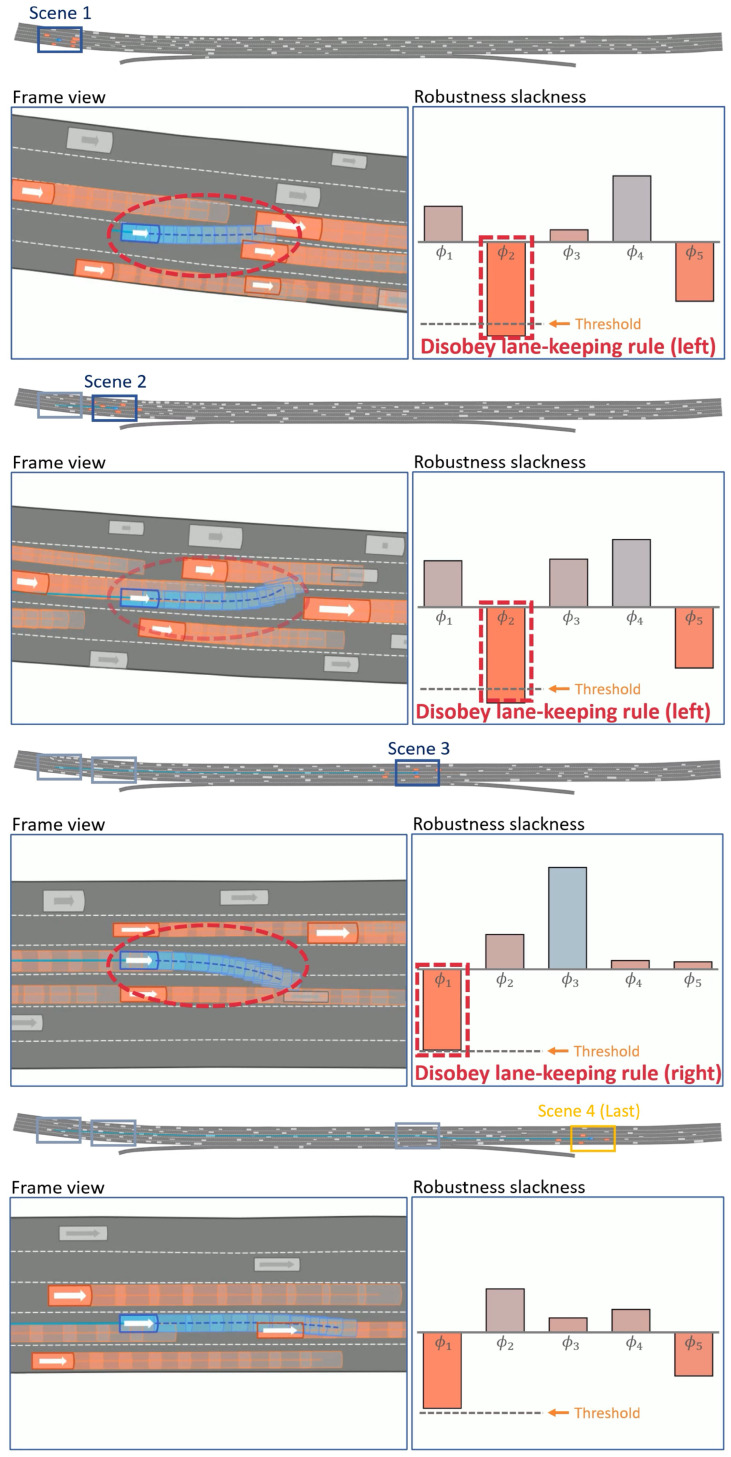
Illustration of the proposed method’s performance in NGSIM road environments. The figure depicts four different scenes, showing the predicted robustness slackness and the corresponding vehicle movements for each situation.

Figure 7 demonstrates the application of the proposed method in the NGSIM road environment. The figure illustrates four different scenes, showing the predicted robustness slackness and the corresponding vehicle movements for each situation. For the lane-keeping rules $\varphi_{1}$ and $\varphi_{2}$, if the robustness slackness value is less than or equal to a specified threshold (indicated by ‘threshold’ in the figure), it is evident that the ego vehicle attempts to change lanes. Conversely, if the robustness slackness value for $\varphi_{1}$ and $\varphi_{2}$ is greater than the threshold value, the proposed method may not initiate a lane change, depending on the specific situation (as illustrated in scene 4). Overall, the proposed method demonstrates the ability to drive efficiently—allowing the violation of some rules in certain situations—while maintaining safety in complex traffic conditions.

Collision experiments using the proposed approach were conducted across five test scenarios: two from the NGSIM dataset and three from the highD dataset. We compared the proposed method against five methods: LBMPC_STL [8], LSTM, TFN [39], and DQN [40]. The LSTM method employs a naive LSTM encoder–decoder framework for imitation learning, while TFN utilizes a Transformer network for imitation learning. In the DQN method, the Q-network is modeled as a four-layer multi-layer perceptron with 12 discrete actions and receives input features. The DQN model was trained until convergence was achieved (1,000,000 episodes).

**Figure 8:**
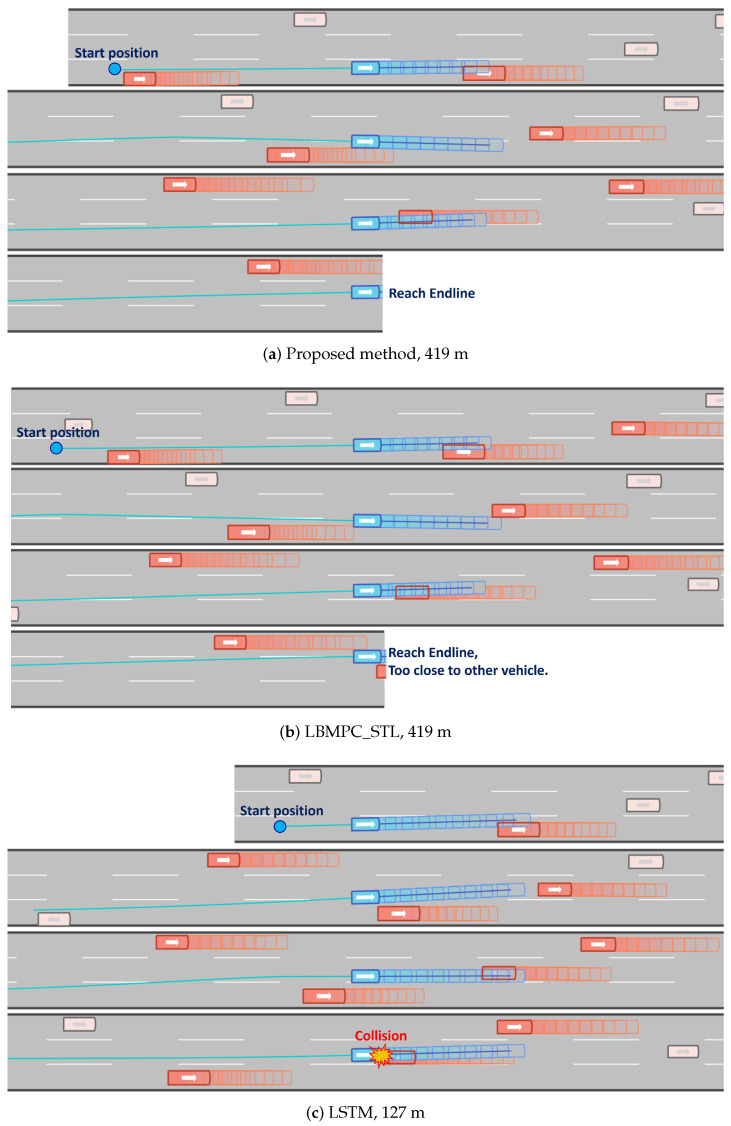
Snapshots of the collision experiments in the highD environment (Proposed, LBMPC_STL, LSTM).

**Figure 9:**
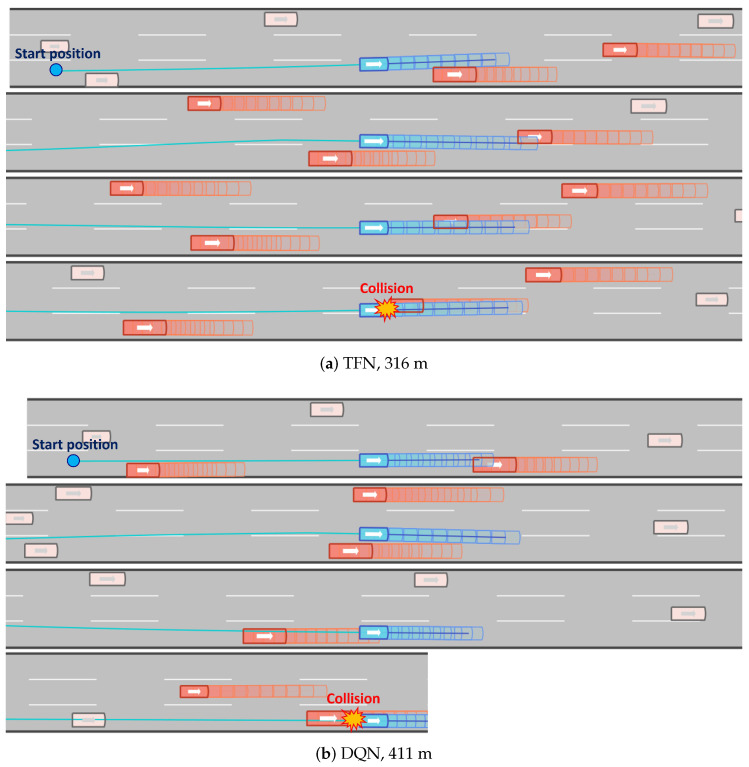
Snapshots of the collision experiments in the highD environment (TFN, DQN).

A total of 100 experiments were conducted on various tracks and starting positions. Figure 8 and Figure 9 illustrate single trials from the collision experiments, demonstrating the performance of each algorithm under identical track conditions, time, and starting positions. The distance traveled by each method is indicated below each subfigure. Methods incorporating MPC (Proposed and LBMPC_STL) successfully arrived at the target area (the end of the track), whereas other methods failed due to collisions. Compared to the proposed method, LBMPC_STL exhibited deficiencies, such as the ego vehicle ending up on the lane border and being too close to the preceding vehicle.

Table 1 presents the number of successful trials for each method. The two methods with the highest number of successes for each scenario are highlighted in bold. The results clearly demonstrate that the proposed approach outperforms other methods in most test scenarios. DQN(1/2) refers to cases where half of the episodes (500,000 episodes) are used in the reinforcement learning stage. The average time steps for successful cases are shown in parentheses.

In the results presented in Table 1, reinforcement learning techniques (specifically DQN) exhibit a longer average time step compared to other methods due to the emphasis on stability in the design of the reward function. Additionally, there was no significant difference in average time steps (for successful cases) between the MPC techniques, including the proposed method, and the supervised learning techniques. Notably, the proposed method demonstrated a slightly shorter average time step compared to the other MPC technique, LBMPC_STL.

While the “average time step” cannot be an absolute criterion for evaluating the superiority of an algorithm’s performance, when combined with the “collision rate”, it indicates that the proposed method enables more stable and efficient autonomous driving compared to other methods.

Two key observations can be made from these results:

- Model Predictive Control (MPC) demonstrates superior safety performance compared to reinforcement learning (DQN) and imitation learning approaches (LSTM, TFN).
- The deep learning approach employed in the proposed method yields a better performance than the Gaussian process regression approach used in LBMPC_STL.

## 7. Conclusions

In this paper, we present a Model Predictive Control (MPC) method designed to manage dynamic systems while adhering to a set of Signal Temporal Logic (STL) rules. Unlike traditional approaches that enforce strict compliance with all rules, our method efficiently balances rule adherence by selectively disobeying certain rules to resolve dilemma situations where not all rules can be simultaneously satisfied.

The proposed method introduces the concept of robustness slackness, which represents the lower bound of the robustness degree, learned from expert demonstrations or data. By employing a Conditional Variational Autoencoder (CVAE) network, the controller adapts its behavior to prioritize different rules based on the context, emulating the decision-making processes of human experts.

Our contribution lies in the innovative approach of learning the satisfaction measure of rules using a deep-learning network, enabling robots to internalize and replicate the value systems of humans. This approach allows for more flexible and context-aware control, which is crucial for operating in complex and dynamic environments.

## Figures and Tables

**Table 1:** Number of successful trials in collision experiments.

	Proposed	LBMPC_STL	LSTM	TFN	DQN	DQN(1/2)
testset 1 (NGSIM-US101)	**88** (180.1)	**84** (189.6)	75 (186.5)	80 (183.4)	82 (199.5)	77 (205.5)
testset 2 (NGSIM-I80)	**86** (175.4)	**81** (180.8)	71 (176.6)	76 (174.2)	79 (187.1)	74 (193.2)
testset 3 (highD)	**89** (124.3)	**86** (129.1)	75 (125.5)	80 (119.9)	83 (147.3)	74 (156.8)
testset 4 (highD)	**91** (131.6)	**90** (131.6)	76 (130.8)	86 (135.2)	88 (148.8)	74 (159.2)
testset 5 (highD)	**95** (136.1)	**93** (139.8)	82 (138.7)	90 (134.3)	91 (159.3)	81 (165.7)

## Data Availability

Data available in a publicly accessible repository.
